# Arbuscular mycorrhizal fungus alleviates anthracnose disease in tea seedlings

**DOI:** 10.3389/fpls.2022.1058092

**Published:** 2023-01-16

**Authors:** Weili Chen, Tao Ye, Qinyu Sun, Tingting Niu, Jiaxia Zhang

**Affiliations:** Tea Research Institute, Anhui Academy of Agricultural Sciences, Huangshan, China

**Keywords:** arbuscular mycorrhizal fungus, anthracnose, tea plant, plant hormone, antioxidant system

## Abstract

Tea has been gaining increasing popularity all over the world in recent years, and its yield and quality depend on the growth and development of tea plants [*Camellia sinensis* (L.) Kuntze] in various environments. Nowadays, biotic stress and extreme weather, such as high temperature, drought, waterlogging, pests, and diseases, bring about much pressure on the production of tea with high quality. Wherein anthracnose, which is the most common and serious disease of tea plants, has earned more and more attention, as its control mainly relies on chemical pesticides. Arbuscular mycorrhizal fungi (AMF), forming symbiosis with most terrestrial plants, participate in plant resistance against the anthracnose disease, which was found by previous studies in a few herbaceous plants. However, there are a few studies about arbuscular mycorrhizal (AM) fungal regulation of the resistance to the anthracnose pathogen in woody plants so far. In this paper, we investigated the effect of AMF on the development of anthracnose caused by *Colletotrichum camelliae* and tried to decipher the pertinent mechanism through transcriptome analysis. Results showed that inoculating AMF significantly reduced the damage of anthracnose on tea seedlings by reducing the lesion area by 35.29% compared to that of the control. The content of superoxide anion and activities of catalase and peroxidase significantly increased (*P* < 0.05) in mycorrhizal treatment in response to the pathogen with 1.23, 2.00, and 1.39 times higher, respectively, than those in the control. Pathways of plant hormone signal transduction, mitogen-activated protein kinase (MAPK) signaling, and phenylpropanoid biosynthesis might play roles in this regulation according to the transcriptomic results. Further redundancy analysis (RDA) and partial least squares structural equation modeling (PLS-SEM) analysis found that plant hormones, such as auxin and ethylene, and the antioxidant system (especially peroxidase) were of great importance in the AM fungal alleviation of anthracnose. Our results preliminarily indicated the mechanisms of enhanced resistance in mycorrhizal tea seedlings to the anthracnose pathogen and provided a theoretical foundation for the application of AMF as one of the biological control methods in tea plantations.

## Introduction

Tea plants [*Camellia sinensis* (L.) Kuntze], originating from Southwest China, are planted in more than 60 countries in tropical and subtropical regions (Tian et al., 2022). Being the third most common nonalcoholic beverage in the world, tea is becoming more and more popular all over the world and has attracted 3 billion drinkers (Pan et al., 2022). Cultivation of tea plants is one of the main ways for farmers to make a living in China and India, which have the most tea plant areas and tea production (Deka and Goswami, 2021). Various biotic and abiotic factors in the environment can affect tea growth and development in many ways. Anthracnose, caused by *Colletotrichum* spp., is one of the most common diseases in tea plants (Wang et al., 2019). Generally, the incidence rate of anthracnose in tea gardens is 5%~8%. The incidence rate in tea gardens seriously affected by anthracnose could reach up to 20%, and the yield of tea will be reduced by more than 30% (Pei, 2012). The anthracnose pathogens adopt a hemi-biotrophic infection strategy to invade tea plant leaves, namely, displaying an initial biotrophic phase and followed by a necrotrophic stage (Jeyaraj et al., 2021). The fungus penetrates tea plant tissues *via* the stomata, hydathodes, or wounds and proliferates in intercellular spaces, especially under warm and humid conditions. To date, chemical pesticides are still the main measure to control the pathogen, which gives pressure on the environment together with the easily developed resistance of the pathogen (Yang et al., 2022). Nowadays, biological control of the disease by utilizing antagonistic bacteria and fungi has been explored as a promising alternative to synthetic fungicides, which obtains increasing interest. Zhou et al. (2021) found that a screened strain of *Trichoderma* spp. increased the resistance to anthracnose of tea seedlings and exerted growth-promoting effects. By screening five tea plant cultivars, five strains of bacteria showed antagonistic effects on the anthracnose pathogen by inhibiting the growth of the hyphae (Chen et al., 2010), which provides a potential way to control the pathogen. Zhou et al. (2022) also confirmed the antifungal action and induction of resistance by *Bacillus* sp. strain YYC 155 against *Colletotrichum fructicola* in *Camellia oleifera*. All of these results made a foundation for the implementation of this environment-friendly way to reduce the damage of anthracnose in tea plantations.

Arbuscular mycorrhizal fungi (AMF), generally forming a symbiotic relationship with approximately 80% of terrestrial plants, can play a crucial role in the plants to face many biotic and abiotic stresses, such as pests, plant pathogens, drought, waterlogging, low temperature, and salinity (Begum et al., 2019; Devi et al., 2022; Sivaprakasam Padmanaban et al., 2022; Zhang et al., 2022). Previous studies demonstrated the function of AMF in controlling anthracnose disease in many herbaceous plants such as *Fragaria×ananassa* Duch. (Matsubara et al., 2006), *Cucumis sativus* L. (Chandanie et al., 2006; Elsharkawy et al., 2012), *Cyclamen persicum* Mill. (Maya et al., 2012), and *Vicia sativa* L. (Ding et al., 2020). Although the antioxidant system (Maya and Matsubara, 2013) and plant hormones, especially jasmonic acid (JA) (Elsharkawy et al., 2012), may be involved in the arbuscular mycorrhizal (AM) fungal regulation on the anthracnose disease based on research on herbaceous plants, the deep mechanisms were not elucidated so far. Compared with herbaceous plants, perennial woody plants have the advantages of developed roots and hard stems and increased secondary xylem, which can enhance biotic and abiotic stress tolerance (Han et al., 2022). However, the effect of AMF on the anthracnose pathogen has not been reported in woody plants.

Therefore, in this paper, we investigated the biocontrol activity of AMF on tea plant seedlings against the anthracnose pathogen *Colletotrichum camelliae*. Transcriptomic analysis was conducted to further decipher the preliminary related mechanisms, aiming to provide a theoretical basis for future biological control of this disease in tea plant cultivation.

## Materials and methods

### Experimental materials

*Rhizophagus intraradices* BGC JX04B was used as the AMF isolate, which was provided by the Beijing Academy of Agriculture and Forestry Sciences (Chen et al., 2017). The inoculum, which consisted of a mixture of spores, mycelium, fine root segments, and growth medium, was prepared by propagating the isolate under clover (*Trifolium repense* L.) as a host for 3 months in the greenhouse.

The strain of *C. camelliae*, isolated from the Anhui tea production area by our laboratory in the Tea Research Institute, Anhui Academy of Agricultural Sciences, was cultured on potato dextrose agar (PDA) at 27°C in darkness for 7 days, which then became the pathogen material used in the next experiment.

Tea plant variety ‘Pingyangtezao’ was used as the plant material in this experiment. Specifically, semi-woody shoots (usually reddish brown) of approximately 5-year-old tea plants under identical growth and management conditions were collected from the tea plant germplasm resource nursery in the Tea Research Institute, Anhui Academy of Agricultural Sciences, Huangshan, China (29°41’18”E, 118°15’39”N). In the laboratory, each shoot was segmented into single node cuttings with approximately 3.5 cm in length. For this study, 300-ml containers were filled with autoclaved (121°C, 2 h) river sand, and 6–7 single-node cuttings were inserted in each container after moistening the cutting medium. All containers were then placed in an artificial climate culture chamber with a light intensity of 5,000 lx and photoperiod of light:dark = 16:8 h. The temperature and relative humidity were 28°C and 80%, respectively. Moreover, 5-g AMF inoculum or sterilized inoculum was added into the river sand in the middle of the container to generate mycorrhizal or non-mycorrhizal seedlings, respectively. To avoid the potential influence of other microbes, we also added 5-ml AM fungal inoculum filtrates (25-μm filter) to the non-mycorrhizal treatment. Moreover, two clover seedlings, as pre-hosts of AMF, were planted in the center of each container.

After 45 days, the mycorrhizal and non-mycorrhizal seedlings of approximately 5-cm height were transplanted into 1-L plastic pots with two tea seedlings in each pot (eight pots in total). The growth substrate (1-kg substrate in each pot) was a mixture of autoclaved (121°C, 2 h) soils and peat (1:1, v:v). As described in the paper of Chen et al. (2021), the soils were collected from the tea plant garden of the Tea Research Institute, Anhui Academy of Agricultural Sciences, Huangshan, China (29°41’18”E, 118°15’33”N), and the soil chemical properties were determined as follows: pH 5.13, organic matter content 5.17 g·kg^−1^, available N 31.40 mg·kg^−1^, available P 2.52 mg·kg^−1^, and available K 76.20 mg·kg^−1^. The substrate was additionally applied with 200 mg·kg^−1^ N [(NH_4_)_2_SO_4_], 100 mg·kg^−1^ K (KNO_3_), and 50 mg·kg^−1^ P (KH_2_PO_4_). All pots were randomly placed in the artificial climate culture chamber, and 50-ml tap water was added to each pot every 3 days. Tea seedlings were grown for 8 months with approximately 20 matured leaves in each seedling, which could be used in further research.

### Experimental design

Firstly, we conducted the pathogenicity test *in vivo* by using one pot of mycorrhizal or non-mycorrhizal tea seedlings as described above. The 7-mm-diameter mycelial or non-inoculated PDA discs were placed on the wounded surface, caused by sterile needles, of the mature leaves (generally the third to fifth leaves). The selected leaves were disinfected in advance with 75% ethanol for 30 s, then rinsed with distilled water and air-dried. PDA discs on leaves were covered by a wet cotton wool to maintain moisture, and the treated pots were placed in the artificial climate culture chamber. Four treatments (three leaves for each treatment) were generated in our study, namely, C-Cc (non-mycorrhizal and non-*C. camelliae* innoculated), C+Cc (non-mycorrhizal and *C. camelliae* innoculated), T-Cc (mycorrhizal and non-*C. camelliae*), and T+Cc (mycorrhizal and *C. camelliae* innoculated). After 3 and 7 days post inoculation (dpi), the vertical and horizontal diameters of lesion areas were measured, respectively, to assess the aggressiveness of the pathogen.

Then, we conducted the second experiment and determined the sampling time at 0 and 3 dpi according to the results of the first experiment. Also, four treatments were generated the same as the first experiment but with three pots in each treatment. Because two sampling times were generated in the second experiment, six treatments were produced in total, namely, C0d, C3d, Cc3d, T0d, T3d, and Tc3d, among which “C,” “d,” “T,” and “c” mean “non-mycorrhizal,” “days post inoculation by the pathogen,” “mycorrhizal,” and “*C. camelliae*,” respectively. Each treatment has three replicates with sampling two leaves from one pot in each replicate. After being photographed for the measurement of the lesion areas by using ImageJ software, the treated leaf samples (another side from the inoculating areas) were taken according to the sampling time and placed in liquid nitrogen for quick freezing and then stored at -80°C. Meanwhile, the shoot and root biomass was weighed at 3 dpi. Then, tea plant roots were freshly kept in 70% ethanol for observing mycorrhizal colonization.

### Determination of mycorrhizal colonization

Mycorrhizal staining was performed according to Phillips and Hayman (1970). Briefly, root segments were incubated with 20% KOH (w/v) in a water bath at 90°C for 30 min, rinsed with tap water, bleached with alkaline hydrogen peroxide (10% H_2_O_2_ + NH_4_OH) for 15 min, acidified in 5% acetic acid for 5 min, and stained with 0.05% Trypan blue in lactoglycerol (lactic acid:glycerol:water, v:v:v = 1:1:1) at 90°C for 60 min. The stained roots were transferred into the destaining solution (lactic acid:glycerol, v:v = 1:1) for 24 h. After that, mycorrhizal colonization was quantified according to Biermann and Linderman (1981).

### RNA sequencing and transcriptomic analysis

Total RNA in the leaf samples from all treatments with three independent replicates in each treatment was extracted using the commercial RNA extraction kit from Accurate Biotechnology Co., Ltd., Hunan, China, according to the manufacturer’s protocol. After that, the RNA integrity was checked with Agilent 2100 Bioanalyzer and Agilent RNA 6000 Nano Kit, and RNA concentration was determined using Denovix DS-11 spectrophotometer (Denovix Inc., USA). When the RNA quality was confirmed, one aliquot was sent to Tsingke Biotechnology Co., Ltd. (Beijing, China), for RNA sequencing (RNA-seq) using the Illumina Hiseq™ 4000 platform and 150-bp paired-end reads were generated. Clean reads were obtained for all subsequent analyses by removing reads containing adapters or more than 5% N (N represents base that could not be determined) and low-quality reads from the raw reads. The *C. sinensis* reference genome (Wei et al., 2018) data, which were downloaded from the Tea Plant Information Archive (TPIA; http://tpdb.shengxin.ren/), were used for the mapping of clean reads by HISAT (Kim et al., 2015). To ascertain the differentially expressed genes (DEGs) between specific treatments, DESeq2 (Love et al., 2014) was performed with Q value ≤0.05 after aligning clean reads to reference sequences by Bowtie2 (Langmead and Salzberg, 2012) and calculating the expression levels of genes by StringTie (Pertea et al., 2015). Gene Ontology (GO) and Kyoto Encyclopedia of Genes and Genomes (KEGG; http://www.genome.jp/kegg/) pathway enrichment analysis of DEGs was implemented by the Blast2GO software (Conesa et al., 2005) and KOBAS 2.0 software (Xie et al., 2011), respectively.

### Quantitative real-time PCR analysis

Another aliquot of extracted RNA was used for the quantitative real-time PCR (qRT-PCR) to validate the RNA-seq results according to a previous protocol (Chen et al., 2021). With the extracted RNA as template, cDNA was synthesized using iScriptTM cDNA Synthesis Kit (Bio-Rad, USA). PCR was conducted with iTaqTM Universal SYBR Green Supermix Kit (Bio-Rad, USA) using LightCycler^®^ 96 Instrument (Roche). The volume of each reaction mixture was 20 μl, containing 5 μl diluted cDNA template, 1 μl 250 nmol·L^−1^ of forward and backward primer, 10 μl SYBR Green supermix, and 4 μl sterilized ddH_2_O. The two-step qRT-PCR was run as follows: 95°C for 30 s, 39 cycles of 95°C for 5 s, and 60°C for 30 s in 96-well plates (Roche). The *18s rRNA* (*AB120309.1*) was selected as a reference gene for qRT-PCR analysis in this paper (Wei et al., 2013). All primers (**Supplementary Table S1**) of randomly selected genes were synthesized in Tsingke Biotechnology Co., Ltd. (Beijing, China). All samples were amplified in triplicate from the same RNA preparation, and the mean values were taken for data analysis. The relative expression of each gene was calculated using the 2^−ΔΔCt^ method (Livak and Schmittgen, 2001).

### Determination of indexes relating to the antioxidant system

All indexes, including the contents of superoxide anion, malondialdehyde (MDA), and hydrogen peroxide (H_2_O_2_), and enzyme activities of catalase (CAT), peroxidase (POD), and superoxide dismutase (SOD) in the plant antioxidant system were analyzed by reagent kits (purchased from Suzhou Comin Biotechnology Co., Ltd.; http://www.cominbio.com/) according to the manufacturer’s procedures.

### Statistical analysis

All data were presented as mean ± standard error of three replicates. For only two treatments, the difference was checked by using independent-sample t-test. Differences among three or more treatments were examined by one-way analysis of variance (ANOVA), and means were compared using Duncan’s multiple-range test. All analyses were carried out through the IBM SPSS v.25 statistical software (SPSS Inc., Chicago, IL, USA).

To find key genes in AM fungal regulation on alleviating the effect of pathogen infection, the correlations between DEGs and lesion indexes, together with antioxidant indexes, were discovered by performing redundancy analysis (RDA) and visualized in CANOCO 5 software (Ter Braak and Šmilauer, 2002). Pearson correlation coefficients were also calculated using SPSS v.25. Additionally, we proposed a hypothetical model by the results of previous studies, which was specified and analyzed with partial least squares structural equation modeling (PLS-SEM) with the support of WarpPLS (version 6.0) software (Kock, 2017), to explore the relationships between AMF inoculation, pathogen infection, biomass, and metabolisms of auxin, ethylene, pathogenesis-related (PR) protein and the antioxidant system.

## Results

### Mycorrhizal colonization and plant biomass

As shown in **Table 1** and **Supplementary Figure S1**, after AMF inoculation, numerous vesicles were found in tea plant roots of mycorrhizal treatment together with the colonization rate of 31.30%, while mycorrhizal colonization was not found in the control. Inoculating AMF promoted the growth of tea plant seedlings. AMF significantly increased the fresh weight of root biomass with a slight decrease of the root/shoot ratio.

**Table 1:** Results of mycorrhizal colonization and plant biomass.

Treatment	Mycorrhizal colonization (%)	Shoot FW (g)	Root FW (g)	R/S
C	0.00 ± 0.00	11.79 ± 1.70	7.19 ± 0.21	0.64 ± 0.11
T	31.30 ± 1.80**	16.41 ± 0.81	10.05 ± 1.79*	0.60 ± 0.04

“*” and “**” indicated significance at P < 0.05 and P < 0.01 in each column, respectively.

C, non-mycorrhizal treatment; T, mycorrhizal treatment; FW, fresh weight; R/S, root biomass/shoot biomass.

### Morphological observation after inoculation with *Colletotrichum camelliae*

In **Supplementary Figure S2**, the lesion size caused by the pathogen *C. camelliae* was smaller in the mycorrhizal treatment (0.55 cm^2^) than that in the control (0.85 cm^2^). Moreover, the area (0.55 cm^2^) and vertical (0.85 cm) and transverse (0.67 cm) diameters of the lesion also significantly decreased (**Table 2**) because of the inoculation with AMF. These results were consistent with the pathogenicity test. Inoculation with AMF increased the tea plant resistant ability to *C. camelliae* as shown in **Supplementary Figure S3** with significantly smaller lesion vertical diameter and transverse diameter compared with those in the control (**Supplementary Table S2**).

**Table 2:** Lesion size induced by AMF after inoculation with Colletotrichum camelliae.

Treatment	Lesion size		
	Area (cm^2^)	Vertical diameter (cm)	Transverse diameter (cm)
**C-Cc**	0.00 ± 0.00 *c*	0.00 ± 0.00 *c*	0.00 ± 0.00 *c*
**C+Cc**	0.85 ± 0.11 *a*	1.04 ± 0.08 *a*	0.87 ± 0.07 d
**T-Cc**	0.00 ± 0.00 *c*	0.00 ± 0.00 *c*	0.00 ± 0.00 *c*
**T+Cc**	0.55 ± 0.02 *b*	0.85 ± 0.01 *b*	0.67 ± 0.03 *b*

Different letters in the same column indicated significance at *P* < 0.05.

C, non-mycorrhizal treatment; T, mycorrhizal treatment; Cc, Colletotrichum camelliae.

### Bioinformatic analysis of RNA sequencing data and identification of differentially expressed genes in tea leaf

RNA-seq analysis was performed to uncover the molecular mechanisms possibly involved in the AM fungal regulation on tea plant resistance to *C. camelliae*. In **Supplementary Table S3**, the overview results of RNA-seq are outlined. Clean reads were obtained, and the proportion of clean Q30 bases was more than 92% (**Supplementary Table S3**) after trimming and applying quality filter. Moreover, more than 82% of these clean reads for each biological repeat were mapped into the *C. sinensis* genome in order to find DEGs in several comparing groups. The RNA-seq dataset, used for analysis in this paper, was deposited in NCBI Short Read Archive (SRA; http://www.ncbi.nlm.nih.gov/sra) under the BioProject of PRJNA883076. Before further analysis, we conducted the validation of the RNA-seq data. A total of 12 genes, which are related to plant hormones, antioxidant system, and phosphorus metabolism, were randomly selected for expression analysis *via* qRT-PCR. The expressions identified by qRT-PCR showed the same trend in general with those observed in RNA-seq (**Supplementary Figure S4**), and there was a significantly positive correlation (*R^2^* = 0.7024, *P* < 0.01, **Supplementary Figure S5**) between RNA-seq and qRT-PCR data, indicating that the RNA-seq data were credible for exploring the mechanism of AM fungal regulation on the resistance of tea plant against anthracnose.

The number of DEG sets in decreasing order was T0d_vs_Tc3d (3,165), C0d_vs_C3d (1,843), T0d_vs_T3d (1,740), C0d_vs_Cc3d (1,696), Cc3d_vs_Tc3d (1,604), T3d_vs_Tc3d (1,367), C3d_vs_T3d (601), C0d_vs_T0d (267), and C3d_vs_Cc3d (242) (**Table 3**). Interestingly, at 3 dpi, the number of DEGs (only 242) in C3d_vs_Cc3d was the lowest, indicating that the control seedlings responded slowly or had low resistance to the pathogen.

**Table 3:** Numbers of differentially expressed genes in various comparing groups.

DEG Set	DEG Number		
	Total	Upregulated	Downregulated
**C0d_vs_C3d**	1,843	842	1,001
**C0d_vs_Cc3d**	1,696	773	923
**C0d_vs_T0d**	267	222	45
**C3d_vs_Cc3d**	242	92	150
**C3d_vs_T3d**	601	119	482
**Cc3d_vs_Tc3d**	1,604	446	1,158
**T0d_vs_T3d**	1,740	656	1,084
**T0d_vs_Tc3d**	3,165	1,444	1,721
**T3d_vs_Tc3d**	1,367	712	655

“DEG”, “C,” “d,” “T,” and “c” indicated “differentially expressed gene,” “non-mycorrhizal,” “days post infection,” “mycorrhizal,” and “*Colletotrichum camelliae*,” respectively.

### Functional classification of differentially expressed genes

All DEGs were assigned to GO categories, and GO terms of each category based on the number of DEGs in all DEG sets are shown in **Supplementary Material 2**. In detail, the most highlighted GO term in all of the sets was “membrane” apart from the set of C0d_vs_C3d. In Molecular Function, GO term “catalytic activity” dominated in all DEG sets, as well as “metabolic process” in Biological Process. Interestingly, except for the set of C0d_vs_T0d, antioxidant activity in other sets was highlighted with the most DEGs in the set of T3d_vs_Tc3d.

Additionally, we also conducted the KEGG pathway analysis, and results are presented in **Supplementary Material 2**. Pathways of MAPK signaling pathway-plant and Plant hormone signal transduction were both important in DEG sets of T3d_vs_Tc3d, T0d_vs_Tc3d, T0d_vs_T3d, Cc3d_vs_Tc3d, and C0d_vs_T0d as shown in cells filled with yellow color in **Supplementary Material 2**. Moreover, the pathway of MAPK signaling pathway-plant also seemed to play a vital role in response to mechanical stress (treated with a needle of a syringe in this paper). These indicated the crucial role of MAPK signaling pathway in both biotic and abiotic stress. In the set of Cc3d_vs_Tc3d, pathways of plant hormone signal transduction (gene ratio of 5.21%), MAPK signaling pathway-plant (4.91%), and phenylpropanoid biosynthesis (4.60%) may participate in the AM fungal regulation on tea plant resistance to the anthracnose pathogen. Pathways of flavonoid biosynthesis (14.52%), carbon metabolism (14.52%), biosynthesis of amino acids (12.90%), starch and sucrose metabolism (9.68%) in the set of C3d_vs_Cc3d may exert a primary function against the pathogen in non-mycorrhizal plant, while in T3d_vs_Tc3d, pathways of carbon metabolism (12.09%), plant hormone signal transduction (9.48%), biosynthesis of amino acids (9.15%), glutathione metabolism (7.52%), and MAPK signaling pathway-plant (6.86%) were important in mycorrhizal plant. Meanwhile, the pathway of phenylpropanoid biosynthesis in both sets was also enriched with many DEGs (**Supplementary Material 2**).

### Differentially expressed genes pertaining to different metabolisms

**Figure 1:**
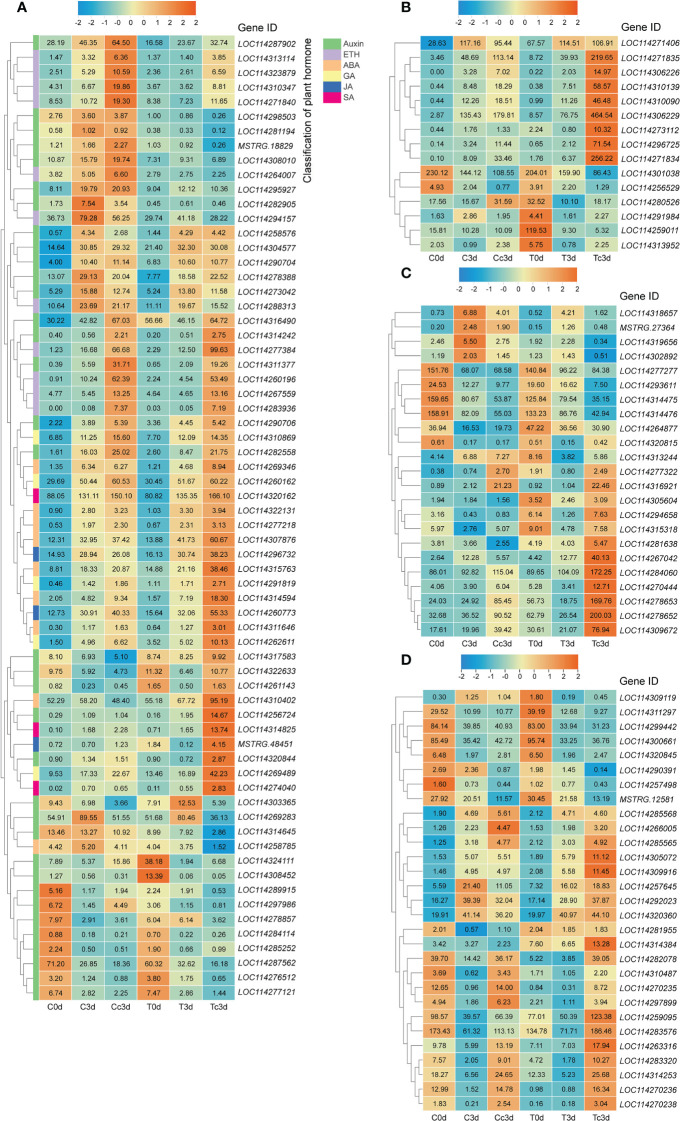
Different categories of differentially expressed genes (DEGs). **(A)** DEGs related to plant hormone. **(B)** DEGs related to pathogenesis-related proteins. **(C)** DEGs related to the antioxidant system. **(D)** DEGs related to flavonoid metabolism. ETH: ethylene; ABA: abscisic acid; GA: gibberellin; JA: jasmonic acid; SA: salicylic acid. “C,” “d,” “T,” and “c” indicated “non-mycorrhizal,” “days post infection,” “mycorrhizal,” and “*Colletotrichum camelliae*,” respectively. The annotation of DEGs is listed in **Supplementary Material 3**.

We selected the DEGs related to the metabolism of the plant hormone, pathogenesis-related proteins, antioxidant system, and flavonoid (**Figure 1**), which are very important in response to the plant pathogens, based on the gene annotations in **Supplementary Material 3**. From all plant hormone-related DEGs, auxin (35 DEGs), ethylene (ETH; 11 DEGs), abscisic acid (ABA; nine DEGs), gibberellin (GA; five DEGs), JA (three DEGs), and salicylic acid (SA; three DEGs) were found to be the potential key hormones (**Figure 1A**), which were possibly involved in AM fungal regulation on tea plant resistance to anthracnose. The 37 DEGs out of 66 DEGs related to plant hormone were upregulated by AMF and anthracnose (Tc3d) compared to other treatments. Interestingly, we found that DEGs related to GA, JA, and SA were all upregulated in mycorrhizal plant together with pathogen infection, especially for JA and SA (**Figure 1A**). In **Figure 1B**, 15 DEGs pertaining to PR proteins were found, and in Tc3d treatment, nearly half of the genes were strongly induced by AMF inoculation when the plant was subjected to the anthracnose pathogen, while this result did not appear in the non-mycorrhizal plant. As for the antioxidant system, 23 DEGs were excavated out and nine DEGs (mainly pertaining to POD) were expressed higher in Tc3d treatment (**Figure 1C**). We also checked out the expression results of DEGs associated with flavonoid metabolism, finding that 21 of 29 genes showed high expressions in mycorrhizal plant leaves induced by pathogen inoculation (**Figure 1D**). In the meantime, there were 15 DEGs that were expressed higher in Cc3d treatment (**Figure 1D**) compared with the mycorrhizal treatment, which may indicate the role of flavonoid metabolism in protecting the control plant against the anthracnose pathogen.

### Contents of malondialdehyde, hydrogen peroxide, and superoxide anion

**Figure 2:**
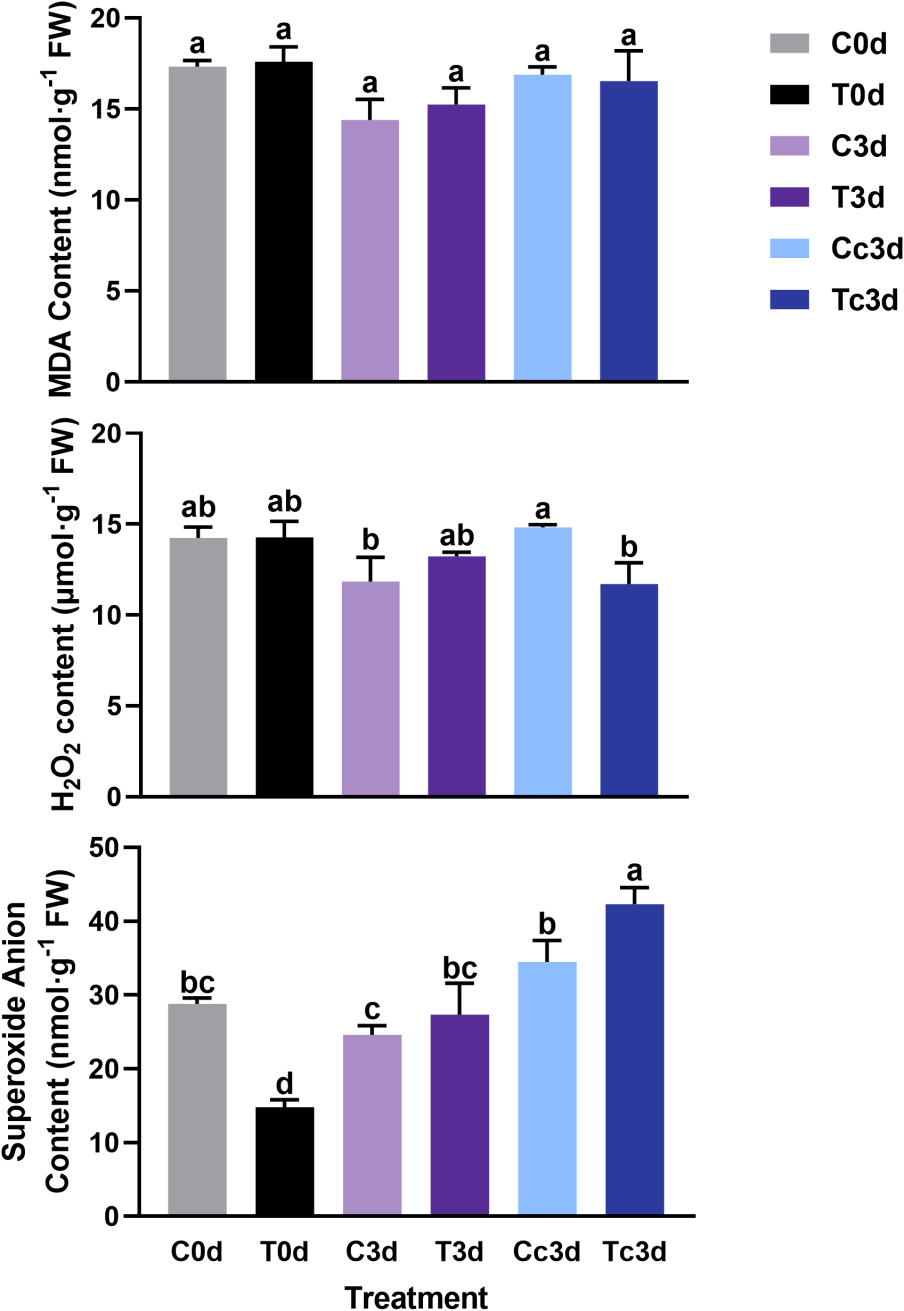
The effect of arbuscular mycorrhizal fungus and anthracnose pathogen on the indexes of active oxygen. H_2_O_2_: hydrogen peroxide; MDA: malondialdehyde. “C,” “d,” “T,” and “c” indicated “non-mycorrhizal,” “days post infection,” “mycorrhizal,” and “*Colletotrichum camelliae*,” respectively. In each column means with similar letters are not significantly different at *p* < 0.05.

As shown in **Figure 2**, there was no significant difference among all six treatments in MDA contents. The H_2_O_2_ content among C0d, T0d, C3d, and T3d also showed no significant difference, while the content was significantly lower in Tc3d (11.71 μmol·g^−1^ FW) than that in Cc3d (14.82 μmol·g^−1^ FW). As for the content of superoxide anion, T0d treatment showed the lowest value (14.80 nmol·g^−1^ FW), which was significantly different from those of the other five treatments. Meanwhile, the treatment of Tc3d (42.31 nmol·g^−1^ FW) showed the highest content of superoxide anion (**Figure 2**).

### Enzyme activities of catalase, peroxidase, and superoxide dismutase

**Figure 3:**
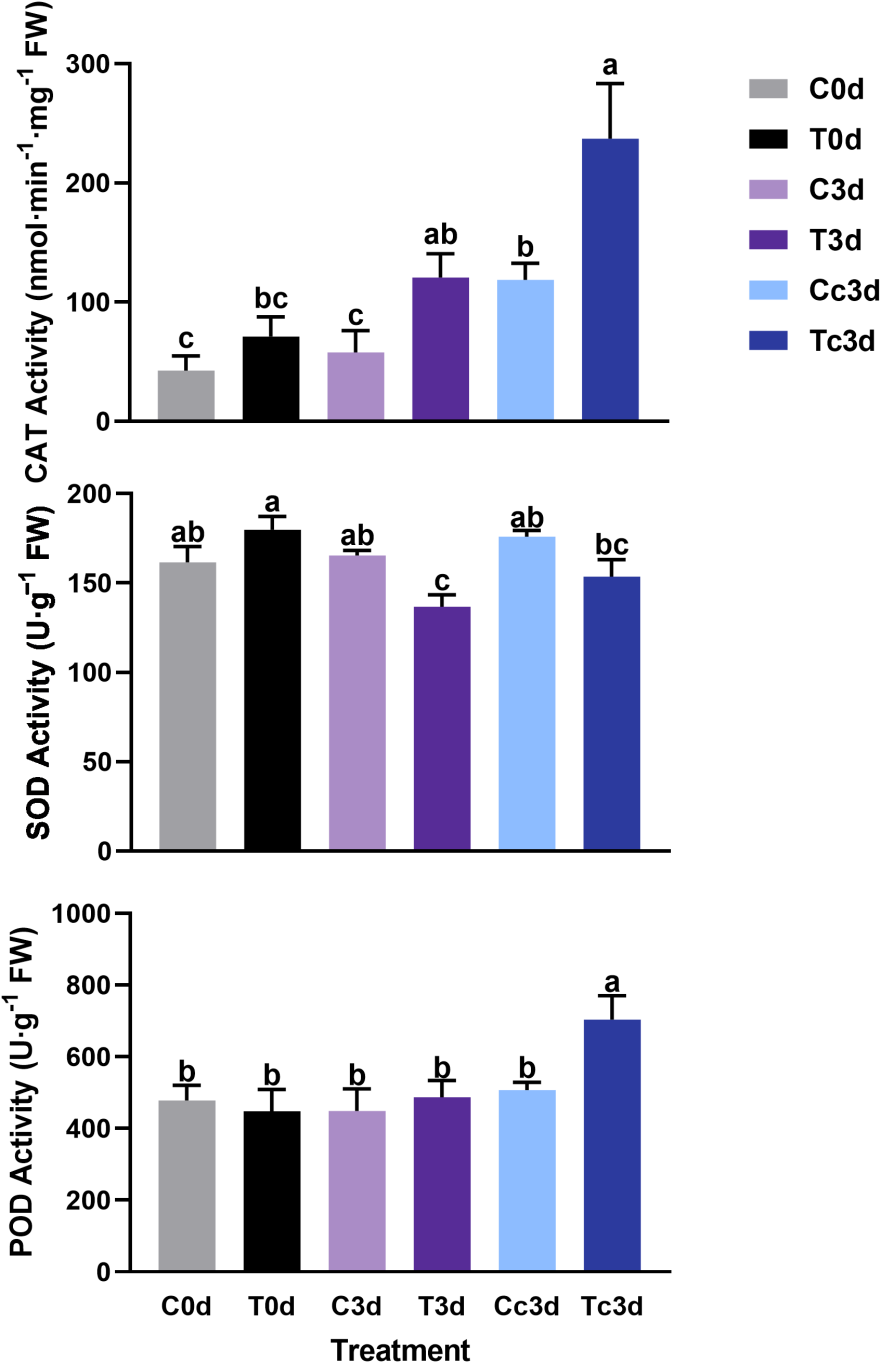
The effect of arbuscular mycorrhizal fungus and anthracnose pathogen on the activities of antioxidant enzymes. CAT: catalase; SOD: superoxide dismutase; POD: peroxidase. “C,” “d,” “T,” and “c” indicated “non-mycorrhizal,” “days post infection,” “mycorrhizal,” and “*Colletotrichum camelliae*,” respectively. In each column means with similar letters are not significantly different at *p* < 0.05.

In **Figure 3**, the treatment of Tc3d showed the highest activities of CAT (237.12 nmol·min^−1^·mg^−1^ FW) and POD (704.05 U·g^−1^ FW), which were both significantly different from other treatments. In treatments of C0d (42.66 nmol·min^−1^·mg^−1^ FW) and C3d (57.87 nmol·min^−1^·mg^−1^ FW), the CAT activities were the lowest. The lowest activity of SOD was found in the treatment of T3d (136.69 U·g^−1^ FW), which was significantly different from those of other treatments, except for Tc3d (153.57 U·g^−1^ FW) (**Figure 3**).

### Correlation analysis of different indexes with the resistance ability to *Colletotrichum camelliae*

**Figure 4:**
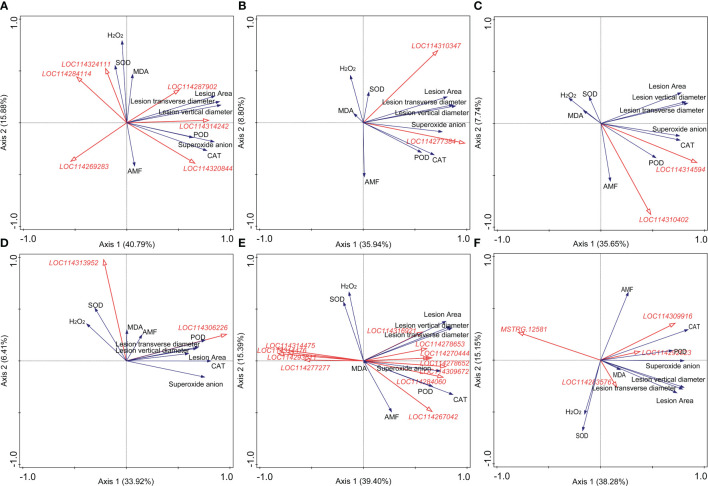
Redundancy analysis (RDA) indicating the correlations between key differentially expressed genes (DEGs) related to different categories and main physiological indexes. **(A)** DEGs related to auxin. **(B)** DEGs related to ethylene. **(C)** DEGs related to abscisic acid. **(D)** DEGs related to pathogenesis-related (PR) proteins. **(E)** DEGs related to the antioxidant system. **(F)** DEGs related to the flavonoid metabolism. AMF: arbuscular mycorrhizal fungus; H2O2: hydrogen peroxide; MDA: malondialdehyde; CAT: catalase; SOD: superoxide dismutase; POD: peroxidase. Blue and red arrows indicated various physiological indexes and key DEGs induced by arbuscular mycorrhizal fungus inoculation and *Colletotrichum camelliae* infection, respectively. The annotation of DEGs in **Figure 5**, filled with yellow, was listed in **Supplementary Material 3**. Physiological indexes included the lesion size, mycorrhizal colonization, reactive oxygen species, and enzymes involved in antioxidant systems.

Results of the correlation analysis and RDA were shown in **Supplementary Material 4** and **Figure 4**, respectively. For plant hormone-related genes, *LOC114269283* (*indole-3-acetic acid-amido synthetase GH3.10*) was significantly negatively correlated with the lesion size, while *LOC114314242* (*auxin-responsive protein IAA26-like isoform X1*), *LOC114310347* (*ethylene receptor 2-like*), *LOC114277384* (*ethylene-responsive transcription factor 1B-like*), *LOC114314594* (*abscisic acid receptor PYL4-like*), and *LOC114310402* (*abscisic acid receptor PYL9-like*) showed positive correlations (**Figures 4A–C**). DEGs related to GA (*LOC114269489*, *LOC114262611*, *LOC114260162*, and *LOC114310869*), JA (*LOC114260773*), and SA (*LOC114320162*), which showed a close correlation with the content of superoxide anion, were all positively correlated with the lesion size (**Supplementary Material 4**). *LOC114306226* (*pathogenesis-related protein PR-4-like*), belonging to the pathogenesis-related proteins, may play a great role in the AM fungal regulation on tea plant resistance to the anthracnose pathogen (**Figure 4D**).

In **Figure 4E**, eight and four DEGs related to the antioxidant system metabolism exerted positive and negative roles in the formation of lesion caused by the pathogen, respectively, indicating the important role of the antioxidant system against the pathogen infection. *LOC114270444* (*peroxidase 17-like*), *LOC114278652* (*lignin-forming anionic peroxidase-like*), *LOC114309672* (*peroxidase 12-like*), *LOC114284060* (*probable L-ascorbate peroxidase 6*), and *LOC114267042* (*peroxidase 73-like isoform X1*) were significantly correlated with the activity of POD (**Supplementary Material 4**). *LOC114293611* (*peroxiredoxin-2E, chloroplastic-like*), *LOC114277277* (*2-Cys peroxiredoxin BAS1, chloroplastic-like*), *LOC114314475* (*peroxiredoxin Q, chloroplastic-like*), and *LOC114314476* (*peroxiredoxin Q, chloroplastic-like*) were all negatively correlated with the lesion size shown in **Figure 4E**. Moreover, combined with the results of correlation analysis and RDA, *MSTRG.12581* (*UDP glucose: flavonoid 3-O-glucosyltransferase*) and *LOC114309916* (*anthocyanidin 3-O-glucosyltransferase 2-like*) were the most significantly related genes to the lesion size in the category of flavonoid metabolism (**Figure 4F**, **Supplementary Material 4**).

### Pathway probing in AM fungal regulation on tea plant resistance to anthracnose

The constructed model could be assumed to be acceptable according to Kock (2017), and the results of “Model fit and quality indices” were presented in **Supplementary Table S4**. For example, the value of Tenenhaus GoF, the widely accepted model fit index for PLS-based path modeling value (Henseler and Sarstedt, 2013), was 0.439, larger than 0.36 (for large effect size), indicating the acceptance of the model.

**Figure 5:**
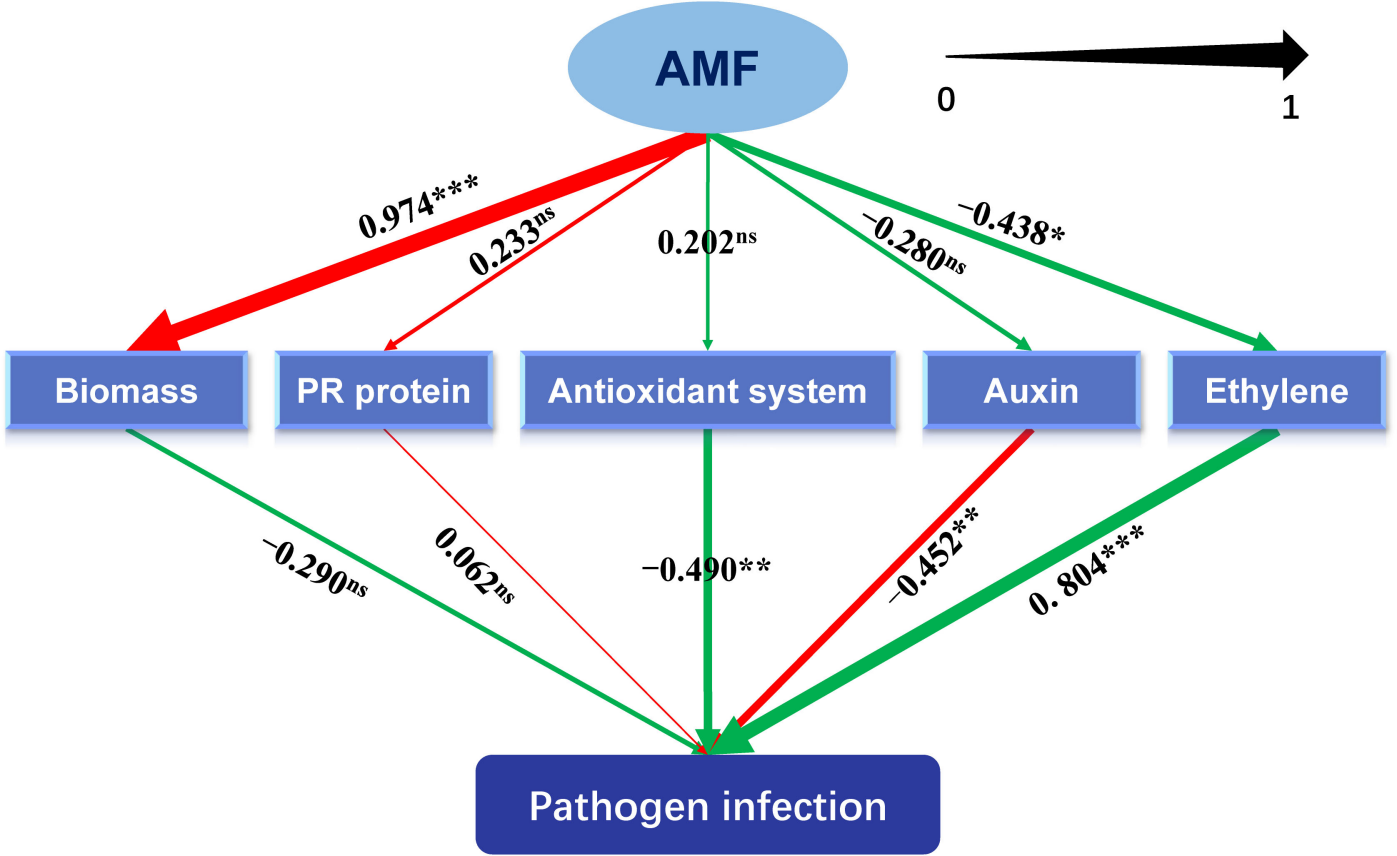
Schematic diagram of the partial least squares structural equation modeling (PLS-SEM). AMF: arbuscular mycorrhizal fungus. Path coefficients were shown and reflected in the width of the arrows. Positive effects are shown as red arrows, negative effects as green arrows. “‘***,” “**,” "*" and “ns” means significance at *p* < 0.001, *p* < 0.01, *p* < 0.05 and non-significance, respectively.

As shown in **Figure 5** and **Supplementary Table S5**, metabolisms related to antioxidant system (-0.490, *P* < 0.01) and auxin (-0.490, *P* < 0.01) significantly inhibited the lesion formation caused by the pathogen, while metabolism related to ethylene (0.804, *P* < 0.001) showed promotive effects. Inoculating with AMF significantly promoted the plant biomass (0.974, *P* < 0.001), which was not exerting an alleviating function on the lesion. The metabolism pertaining to ethylene was negatively regulated by AMF (-0.438, *P* < 0.05). Moreover, the total effect (-0.593, *P* < 0.001) of AMF on the formation of lesion was significantly negative.

## Discussion

### Alleviated hypersensitive necrotic reaction and highly expressed pathogenesis-related proteins by AMF

Hypersensitive necrotic reaction (HR), one of the plant active resistance ways that can be actively expressed in response to biotic stresses, is a phenomenon of programmed cell death (PCD). The occurrence of HR prevents the further invasion of the plant pathogen, which then protects the plant. Generally, HR is associated with the activation of nucleotide-binding site–leucine-rich repeat (NBS-LRR) proteins by pathogen effectors, and the NBS-LRRs act together with multiple defense-related genes in plants to defend against *Colletotrichum* spp. (Wang et al., 2018). In our results, HR was quantified with the size of the lesion, and in mycorrhizal tea seedling, the lesion size, caused by *C. camelliae*, was apparently small (**Supplementary Figure S2**, **Table 2**), indicating the reduced severity of anthracnose that was consistent with the study on *V. sativa* L. (Ding et al., 2020) and *C. persicum* Mill. (Maya and Matsubara, 2013). In these plants, the growth promotion by AMF was also found, which may be related to the enhanced resistance to anthracnose, because of a slightly negative correlation between biomass and pathogen infection in the analysis of PLS-SEM (**Figure 5**). It could be speculated that well growth state may be one of the contributors for plants being armed with more induced resistance to pathogens, which needs more evidence in the future.

During HR, the expression of PR proteins will increase in infected plants. PR proteins are the key proteins for the formation of acquired resistance, which are directly correlated with plant resistance (Joshi et al., 2021). PR proteins, produced by *PR* genes after responding to pathogen invasion signals, are acidic and secretory small-molecule proteins with antimicrobial abilities, which develop antifungal activity mainly in the cell wall by direct contact or hydrolysis of pathogen cell wall (Selitrennikoff, 2001; Fu and Dong, 2013). Many studies about the performance of PR proteins in plant resistance to different pathogens can be found. *PR-1* genes were found from *Piper nigrum* L. and displayed a critical role during *Phytophthora capsici* infection (Kattupalli et al., 2021). AMF alleviated *Medicago sativa* leaf spots caused by *Phoma medicaginis*, in which genes belonging to PR proteins participated (Li et al., 2021a). Our results found that 15 DEGs related to PR proteins were induced and eight of 15 DEGs were highly expressed in Tc3d treatment, indicating the important role of these proteins in AM fungal regulation on the anthracnose pathogen, although there were still some PR proteins that showed opposite results (**Figure 1B**). Similar results were also found in the study on herbaceous plant, *V. sativa* L. (Ding et al., 2020). Fu and Dong (2013) found that it is difficult to genetically test the contribution of each PR protein to pathogen resistance because these proteins are likely to work in concert and are often encoded by multiple genes in gene clusters. Interestingly, the PLS-SEM results showed that the pathway of PR proteins may not participate in the AM fungal induced resistance (**Figure 5**). Further evidence is needed to confirm the direct or indirect way of PR proteins in response to the anthracnose pathogen after inoculating with AMF.

### Reactive oxygen species burst and enhanced antioxidant system in mycorrhizal tea plant leaves induced by anthracnose

One of the earliest plant responses to pathogen infection is the reactive oxygen species (ROS) burst. The function of ROS, including superoxide anion, H_2_O_2_, and hydroxyl radicals, in the plant resistance to pathogen mainly consists of direct antimicrobial ability, lignification of the cell wall, induction of HR, production of PR proteins, generation of phytoalexins (like flavonoid), and other defense responses (Shetty et al., 2008; Li et al., 2021b; Feng et al., 2022). H_2_O_2_, acting as a signal, can activate downstream defense responses, and recent studies showed that H_2_O_2_ signal could be transduced to aquaporins to endow a resistant ability against pest (Lu et al., 2022) and disease (Zhang et al., 2021) in plant. In this paper, the content of superoxide anion was significantly higher in Tc3d than that in other treatments, while the content of H_2_O_2_ was the lowest together with the C3d treatment. The results may be due to the low activity of SOD, which catalyzes the dismutation of superoxide radicals to molecular oxygen and H_2_O_2_, thus providing cellular defense against ROS (Su et al., 2021). Different from SOD, POD was strongly induced in Tc3d, and POD-related DEGs were also highly expressed, indicating its crucial role in the resistance triggered by AMF. Bi et al. (2022) also found that the cytosolic thiol peroxidase PRXIIB is an intracellular sensor for H_2_O_2_ that can regulate plant immunity against *Pseudomonas syringae* in *Arabidopsis thaliana*, in which PRXIIB conjugates *via* Cys51 with the type 2C protein phosphatase ABA insensitive 2 (ABI2), subsequently transducing H_2_O_2_ signal to ABI2. Moreover, the activity of CAT showed a similar pattern with that of POD. These two enzymes show a similar function of scavenging H_2_O_2_ (Naliwajski and Skłodowska, 2021). Contrary to our results, increased SOD activities were shown in the study on *C. persicum* Mill. (Matsubara et al., 2013; Maya and Matsubara, 2013) and *V. sativa* L. (Ding et al., 2020). However, the elevated POD activity was found in the latter compared with our results. We speculated that these were possibly related to the plant species or sampling time. In our results, AMF significantly induce the antioxidant system revealed by the PLS-SEM analysis (**Figure 5**), and the RDA results also underlined their importance in lesion formation (**Figure 4E**). Taken together, the pathway of the antioxidant system may play a great role in tea plant resistance to anthracnose pathogen, which was enhanced by AMF inoculation. Additionally, approximately 29 DEGs related to flavonoid metabolism (**Figure 1D**), which also can alleviate the reactive oxygen damage (Shen et al., 2022), were upregulated by AMF, although the RDA (**Figure 4F**) showed that only five DEGs may be the most important genes involved in resistance to the pathogen. The pathway of flavonoid biosynthesis is important in AM fungal regulation on plant resistance to disease. Rashad et al. (2020) found that accumulations of chlorogenic acid, flavonoids, and anthocyanins triggered by *R. irregularis* were related to the reduced disease severity in *Helianthus annuus* L., and Aseel et al. (2019) also found that AMF helped *Solanum lycopersicum* L. against tomato mosaic virus through the elevated expressions of genes in flavonoid biosynthetic pathways. Additionally, a previous study had indicated that flavonoid metabolism was involved in *C. oleifera* defense against anthracnose (Yang et al., 2022), and Yin et al. (2022) found that overexpression of *vqWRKY31* enhances powdery mildew resistance in *V. vinifera* L. by promoting flavonoid synthesis. Therefore, it is a necessity of further study by metabolome and other methods to confirm the flavonoid’s role in the effect of AMF on the resistance to anthracnose pathogen and relative mechanism.

### Plant hormone metabolism in AM fungal regulation on resistance to anthracnose

Plant hormones are complex and important signaling molecules that modulate many aspects of plant development and defense (Shan et al., 2012). Our KEGG enrichment results indicated that the pathway of plant hormone signal transduction may play a key role in the tea plant resistance to *C. camelliae* (**Supplementary Material 3**). Firstly, the plant hormone auxin governs many aspects of normal plant growth and development. Auxin also plays an important role in plant–microbe interactions, including interactions between plant hosts and pathogenic microorganisms, which cause disease (Kunkel and Johnson, 2021). In general, indole-3-acetate (IAA) can act both as a plant hormone that modulates host signaling and physiology to increase host susceptibility and as a microbial signal that directly impacts the pathogen by producing virulence (Svoboda et al., 2021). A previous study has revealed that IAA biosynthesis may participate in the tea plant–*C. camelliae* interaction (Lu et al., 2020). In our study, the biggest number of auxin-related DEGs, accounting for more than 50% of all DEGs pertaining to plant hormones (**Figure 1A**, **Supplementary Material 3**), was found among all treatments, and we screened two DEGs, *LOC114269283* (*indole-3-acetic acid-amido synthetase GH3.10*) and *LOC114314242* (*auxin-responsive protein IAA26-like isoform X1*), which were the most significantly correlated genes with lesion size shown in RDA (**Figure 4A**). In order to clarify the role of auxin in AMF-induced resistance, we conducted the PLS-SEM analysis and found that auxin may promote lesion formation (**Figure 5**).

Ethylene is an important phytohormone in mediating defense genes and proteins (especially for ethylene-responsive factors), and we found a significantly positive correlation between ethylene and pathogen infection, which was induced by AMF (**Figure 5**). Most DEGs of ethylene-responsive factors were upregulated in the double inoculation of AMF and *C. camelliae*, possibly implying that the transcription factors were one of the key pathways to resist the pathogen after AMF inoculation. Ethylene-responsive factors are excellent candidates for developing plant resistance to anthracnose and other fungal diseases (Svoboda et al., 2021), and loss of ERF114 function caused impaired disease resistance (Li et al., 2022). Therefore, future research could be focused on this field. ABA, a sesquiterpenoid, also acts as a signaling molecule mediating plant adaptation to environmental stresses (Svoboda et al., 2021). Expressions of *abscisic acid receptor PYL4-like* and *abscisic acid receptor PYL9-like* were strongly induced by AMF in tea plant leaves when infected by anthracnose pathogen (**Figure 4B**, **Supplementary Material 3**), which are also found in other studies (Fu et al., 2022; Liang et al., 2022; Zheng et al., 2022).

Additionally, five DEGs related to GAs, which also function in plant defense system (Bari and Jones, 2009), were all positively correlated with the lesion size (**Supplementary Material 4**). *Gibberellin receptor GID1* genes are important for preventing twig blight pathogen in bayberry (Ren et al., 2021), indicating the positive role in reducing damages of the plant pathogens. According to previous reports (Alvarez, 2000; Vergne et al., 2010; Lu et al., 2013), SA is involved in the resistance induced by biotrophs and hemibotrophs with the occurrence of systematic acquired resistance (SAR), while JA participates in the resistance from necrotrophs and pests by activating the induced systemic resistance (ISR). The anthracnose pathogen belongs to hemibotrophs, and we found that both SA- and JA-related DEGs were involved in the AM fungal regulation on the tea plant resistance (**Supplementary Material 4**). This was evidenced by another study on banana fruit, which found that application of exogenous SA and methyl jasmonate (MeJA) induced resistance of banana fruit to anthracnose disease through activating PR proteins and mitogen-activated protein kinase (MAPK) transcription factors (Tang et al., 2013). However, Zhu et al. (2019) only studied that the application of exogenous SA stimulated the high expression of both *CsKPI2* and *CsKPI3* (Kunitz protease inhibitors) to confer resistance to anthracnose in tea plant. So, more studies should be carried out to prove the role of SA and JA in tea plant resistance to anthracnose pathogen.

## Conclusion

As previous studies of AM fungal regulation on resistance to anthracnose pathogen were mainly conducted in herbaceous plants, in this paper, we investigated whether the resistance of the woody plant, tea plant, to *C. camelliae* will be affected by inoculating AMF. The results demonstrated the inhibition of lesion size by AMF, indicating the elevated resistance to the pathogen, and we also preliminarily revealed the related mechanism in this interaction. Pathways of plant hormone (especially auxin and ethylene) and antioxidant system may play key roles, and POD might be the most important enzyme in AM fungal alleviation of anthracnose. However, deeper work should be done to underpin the mechanism in the future.

## Data availability statement

The datasets presented in this study can be found in online repositories. The names of the repository/repositories and accession number(s) can be found in the article/**Supplementary Material**.

## Author contributions

WC and JZ conceived and designed the manuscript, and WC wrote the manuscript. TN, TY, and QS provided great help in the whole experiment. All authors approved the manuscript for submission.
