# Supplementary material for: Arbuscular mycorrhizal fungus alleviates anthracnose disease in tea seedlings
Source: Front Plant Sci. 2023 Jan 16;13:1058092. doi: 10.3389/fpls.2022.1058092 (PMC9886063; doi:10.3389/fpls.2022.1058092)

Supplementary Material

Title:

Arbuscular mycorrhizal fungus alleviates anthracnose disease in tea seedlings

Authors:

Weili Chen, Tao Ye, Qinyu Sun, Tingting Niu, Jiaxia Zhang*

*****Correspondence: Jiaxia Zhang: [zhangjiaxia035@163.com](mailto:zhangjiaxia035@163.com)

## Supplementary Tables and Figures

**Supplementary Table S1** Primers of each gene in real-time quantitative RT-PCR

| **Gene ID** | **Gene Description** | **Sequence of primers (5’-3’)** |
| --- | --- | --- |
| AB120309.1 | Camellia sinensis 18S Ribosomal RNA | F: CGCGCAAATTACCCAATCCT |
|  |  | R: ACCAGACTTGCCCTCCAATG |
| LOC114261524 | ethylene-responsive transcription factor ERF113-like isoform X1 | F: CACGTCGATGGGACAACAAG |
|  |  | R: ACTTGTGGAAGAGGGCGAAA |
| LOC114279627 | transcription factor MYB15-like | F: ACCCAGTTGTCCAAGTCCAAA |
|  |  | R: CCTTCAACTCACCACTCGGG |
| MSTRG.28700 | disease resistance protein RPP13-like isoform X1 | F: TTGGTTGCTGTGCAGGGATA |
|  |  | R: ATTTCCTTGCGGACACCCTT |
| LOC114306662 | F-box/LRR-repeat protein At3g59190-like isoform X1 | F: AGCTCTTTTCTTCCTGCCCTG |
|  |  | R: AGAGTTTCGGTATTGCGTCG |
| LOC114323824 | abscisic acid 8'-hydroxylase CYP707A2-like | F: ATAGTGAGAGAGAAGCGCGG |
|  |  | R: TCTGGGCTGTGATGGATGTTT |
| LOC114282905 | auxin-induced protein 22D-like | F: CCAGACCTACCCTGAGCTACT |
|  |  | R: AGTAGGTGTGTAGTTGCGCT |
| LOC114256724 | probable indole-3-acetic acid-amido synthetase GH3.1 | F: CGTTCAAGTCAAGAGTGCCG |
|  |  | R: TACAGTAGCTGCCTTCGGTC |
| LOC114267042 | peroxidase 73-like isoform X1 | F: GCTGAGTTCAACAGCTGCAAG |
|  |  | R: GAGTAGGGTCCACTGGGTTT |
| LOC114276797 | inorganic phosphate transporter 1-4-like | F: GGTGCCTGAGTCGAAAGGAA |
|  |  | R: TGTCATGACTGGTCTGCTGTC |
| LOC114314522 | phosphoenolpyruvate/phosphate translocator 2, chloroplastic-like, partial | F: TGAGAATCTAGGTGGTGTTTCAGAT |
|  |  | R: CCTCCCTCCAATGAAAGCGG |
| LOC114275394 | pyrophosphate--fructose 6-phosphate 1-phosphotransferase subunit beta-like isoform X1 | F: AAGAATGGACAGTGGGTGGC |
|  |  | R: AAACTGAAGTGGGCCTGGAC |
| LOC114314825 | salicylic acid-binding protein 2-like isoform X1 | F: CCCCAGAAAATCTCCACTGC |
|  |  | R: GTCCAACCATGATTCTGGGG |

**Supplementary Table S2** Effects of AMF on lesion size induced by inoculating with *Colletotrichum camelliae*.

| Treatment | 3 d | | 7 d | |
| --- | --- | --- | --- | --- |
|  | Vertical  diameter (cm) | Transverse  diameter (cm) | Vertical  diameter (cm) | Transverse  diameter (cm) |
| C-Cc | 0.00±0.00c | 0.00±0.00c | 0.00±0.00c | 0.00±0.00c |
| C+Cc | 1.04±0.08a | 0.87±0.07a | 2.00±0.10a | 1.82±0.07a |
| T-Cc | 0.00±0.00c | 0.00±0.00c | 0.00±0.00c | 0.00±0.00c |
| T+Cc | 0.85±0.01b | 0.67±0.03b | 1.74±0.16b | 1.50±0.14b |

Different letters in the same column indicated significance at *P* < 0.05; C: non-mycorrhizal treatment; T: mycorrhizal treatment; Cc: *Colletotrichum camelliae*.

**Supplementary Table S3** Main results of RNA-Seq.

| Treatments | Clean reads | ≥Q30 clean bases (%) | GC Content （%） | Mapped Reads | Unique Mapped Reads | Mapping ratio (%) |
| --- | --- | --- | --- | --- | --- | --- |
| C0d1 | 21,186,083 | 92.92 | 44.55 | 38,837,059 | 36,354,152 | 85.80 |
| C0d2 | 21,548,182 | 92.99 | 45.76 | 39,902,232 | 37,086,258 | 86.05 |
| C0d3 | 18,809,959 | 93.22 | 46.43 | 34,651,343 | 32,025,844 | 85.13 |
| C3d1 | 27,867,330 | 93.00 | 44.64 | 51,073,025 | 47,629,624 | 85.46 |
| C3d2 | 21,968,889 | 92.88 | 44.21 | 40,130,647 | 37,478,559 | 85.30 |
| C3d3 | 28,913,924 | 93.18 | 46.30 | 53,479,278 | 48,873,028 | 84.51 |
| Cc3d1 | 21,618,106 | 93.67 | 44.26 | 39,593,424 | 36,984,019 | 85.54 |
| Cc3d2 | 21,157,730 | 93.44 | 44.41 | 38,811,425 | 36,185,783 | 85.51 |
| Cc3d3 | 20,682,023 | 92.90 | 46.24 | 38,167,869 | 35,106,687 | 84.87 |
| T0d1 | 19,486,494 | 93.28 | 45.46 | 35,992,222 | 33,449,621 | 85.83 |
| T0d2 | 20,601,030 | 93.73 | 45.75 | 38,047,409 | 35,262,169 | 85.58 |
| T0d3 | 22,790,906 | 93.20 | 45.83 | 42,135,617 | 38,857,467 | 85.25 |
| T3d1 | 20,562,150 | 93.46 | 45.83 | 38,028,816 | 35,126,775 | 85.42 |
| T3d2 | 21,436,146 | 93.40 | 45.42 | 38,555,409 | 35,577,044 | 82.98 |
| T3d3 | 20,657,756 | 94.30 | 45.74 | 38,054,651 | 35,158,337 | 85.10 |
| Tc3d1 | 24,856,745 | 93.35 | 46.02 | 45,848,050 | 41,851,767 | 84.19 |
| Tc3d2 | 20,594,665 | 93.27 | 45.34 | 38,046,164 | 35,135,667 | 85.30 |
| Tc3d3 | 22,948,866 | 94.67 | 46.23 | 42,262,930 | 38,496,091 | 83.87 |

C: non-mycorrhizal treatment; T: mycorrhizal treatment; c: *Colletotrichum camelliae*; d: days post infection.

**Supplementary Table S4** Model fit and quality indices.

| **Index** | **Value** | **Value Interpretation** |
| --- | --- | --- |
| Average path coefficient (APC) | 0.423  *p =* 0.008 | Significant if *p* <  0.05 |
| R-squared (AARS) | 0.367  *p =* 0.016 | Significant if *p* <  0.05 |
| Average adjusted R-squared (AARS) | 0.320  *p =* 0.028 | Significant if *p* <  0.05 |
| Average block VIF (AVIF) | 1.240 | acceptable if <= 5, ideally <= 3.3 |
| Average full collinearity VIF (AFVIF) | 3.196 | acceptable if <= 5, ideally <= 3.3 |
| Tenenhaus GoF (GoF) | 0.439 | small >= 0.1, medium >= 0.25, large >= 0.36 |
| Sympson’s paradox ratio (SPR) | 1.000 | acceptable if >= 0.7, ideally = 1 |
| Statistical suppression ratio (SSR) | 0.800 | acceptable if >= 0.7 |
| R-squared contribution ratio (RSCR) | 1.000 | acceptable if >= 0.9, ideally = 1 |

**Supplementary Table S5** Total effects of different paths.

| **Path** | **Path coefficient** | ***p* value** |
| --- | --- | --- |
| AMF→Biomass | 0.974 | <0.001 |
| AMF→PR | 0.233 | 0.133 |
| AMF→Antioxidant system | 0.202 | 0.171 |
| AMF→Auxin | -0.280 | 0.086 |
| AMF→Ethylene | -0.438 | 0.012 |
| Biomass→Pathogen infection | -0.290 | 0.078 |
| PR→Pathogen infection | 0.062 | 0.394 |
| Antioxidant system→Pathogen infection | -0.490 | 0.006 |
| Auxin→Pathogen infection | -0.452 | 0.010 |
| Ethylene→Pathogen infection | 0.804 | <0.001 |
| AMF→Pathogen infection  (Total effect) | -0.593 | <0.001 |

**Supplementary Figure S1** The observation of mycorrhizal colonization in tea plant roots. C: non-mycorrhizal treatment; T: mycorrhizal treatment


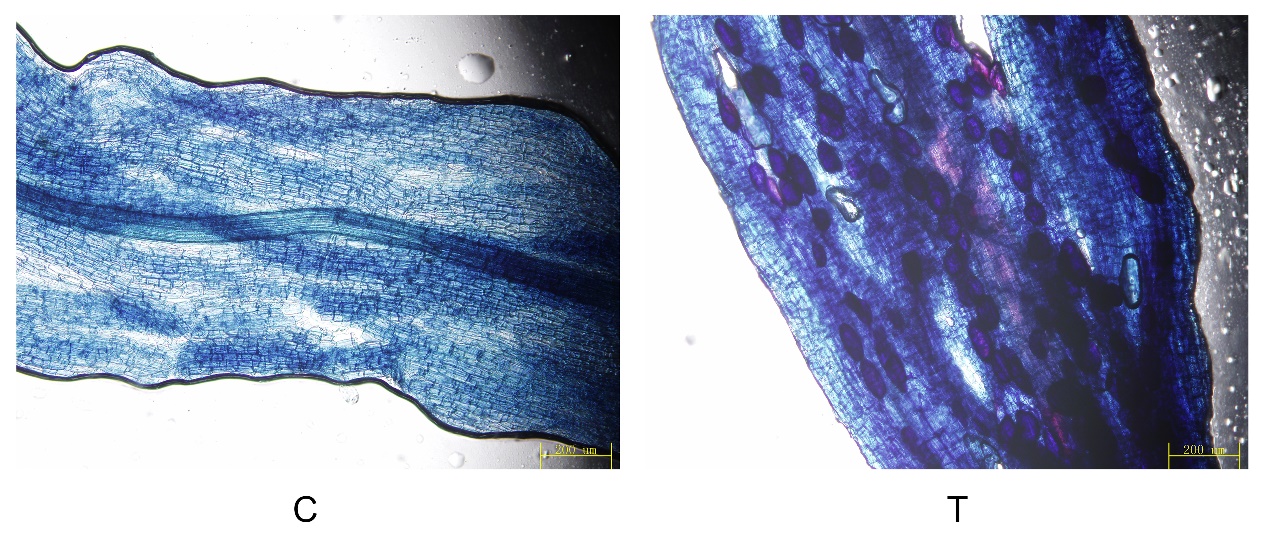


**Supplementary Figure S2** Assessment of anthracnose on tea seedlings in different treatments at 3 days post infection. C: non-mycorrhizal treatment; T: mycorrhizal treatment; Cc: *Colletotrichum camelliae*.


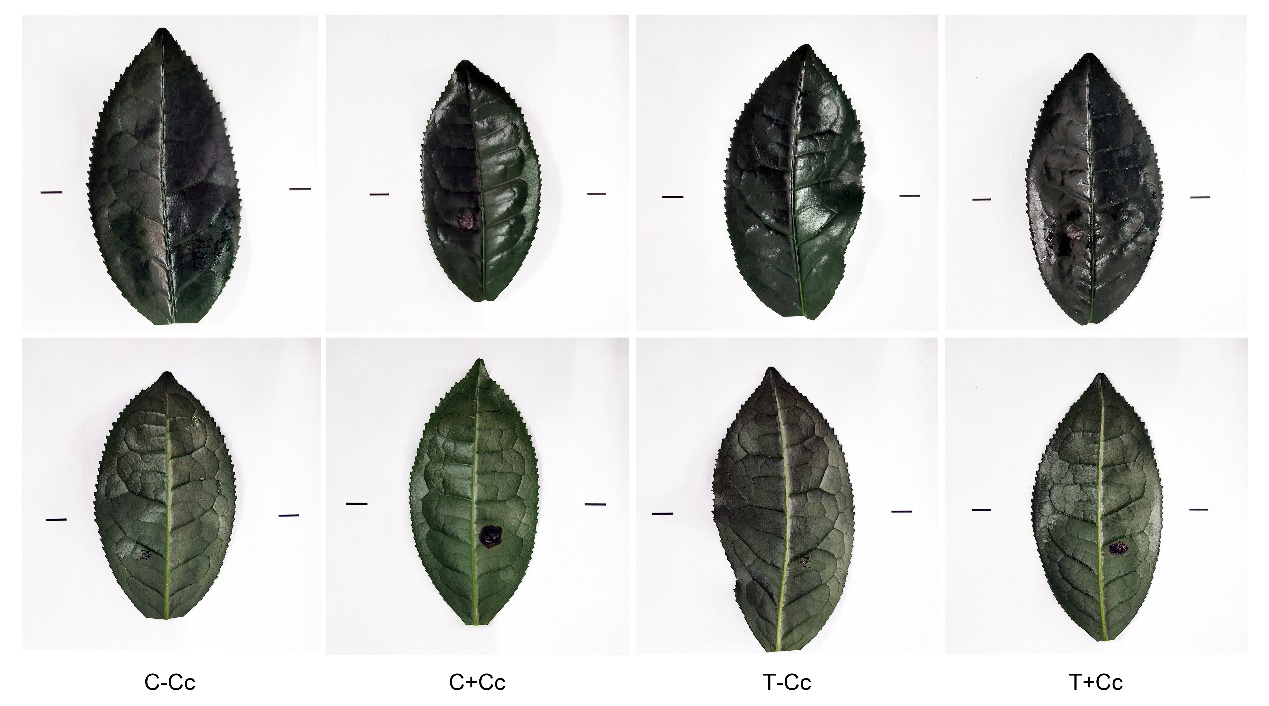


**Supplementary Figure S3** The size of leaf lesions after 3 and 7 days inoculating with *Colletotrichum camelliae*. C-Cc: non-mycorrhizal and non-*Colletotrichum camelliae*; C+Cc: non-mycorrhizal and inoculating *Colletotrichum camelliae*; T-Cc: mycorrhizal and non-*Colletotrichum camelliae*; T+Cc: mycorrhizal and inoculating *Colletotrichum camelliae*. dpi: days post inoculated.


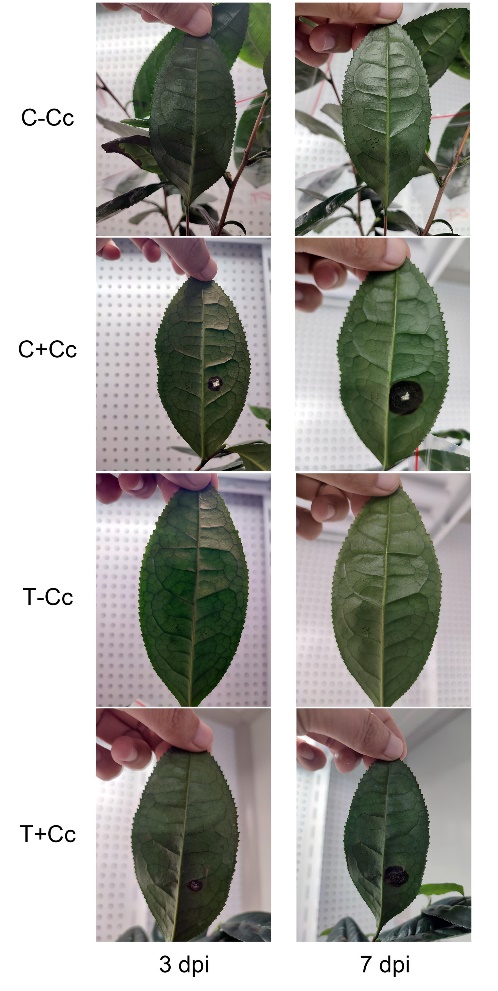


**Supplementary Figure S4** Validation of RNA-seq results by qRT-PCR. Data from qRT-PCR (Black-filled circle) and RNA-Seq (White column) of 12 selected genes were means of three replicates and bars represent Standard Error. C: non-mycorrhizal treatment, T: mycorrhizal treatment.


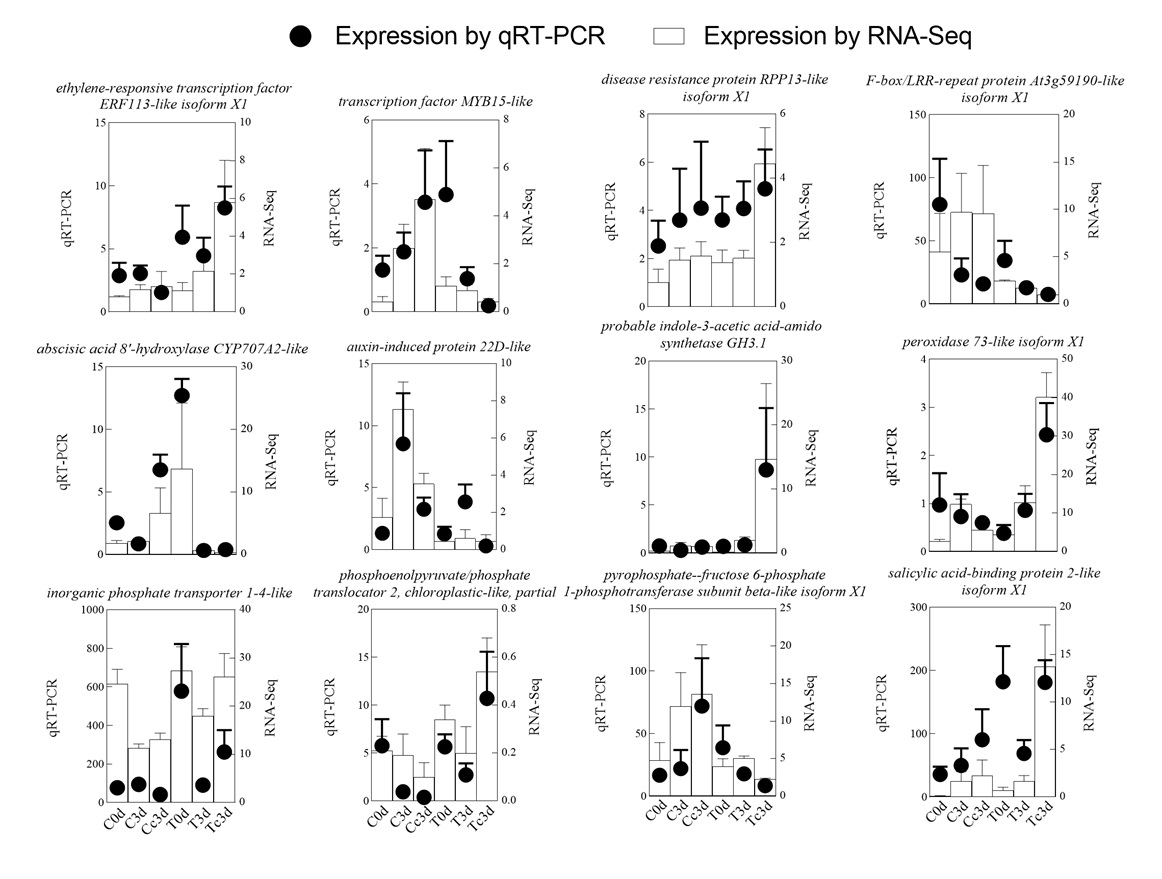


**Supplementary Figure S5** Correlation analysis of fold change data between qRT-PCR and RNA-seq. Scatterplots were generated by the log2(Fold change) from RNA-seq (x-axis) and qRT-PCR (y-axis).


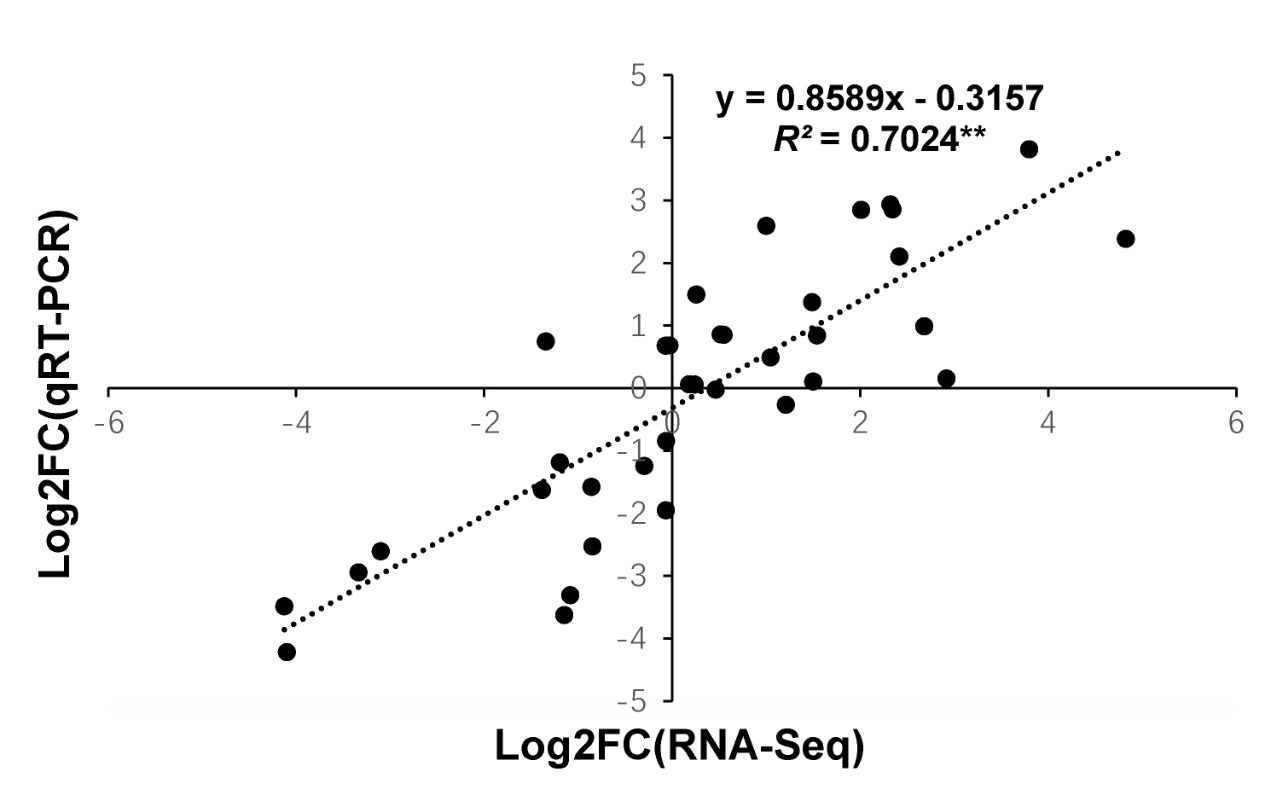

Supplement: Supplementary file 1 [file DataSheet_1.docx]
